# A falls prevention programme to improve quality of life, physical function and falls efficacy in older people receiving home help services: study protocol for a randomised controlled trial

**DOI:** 10.1186/s12913-017-2516-5

**Published:** 2017-08-14

**Authors:** Maria Bjerk, Therese Brovold, Dawn A. Skelton, Astrid Bergland

**Affiliations:** 1 Department of Physiotherapy, Faculty of Health Sciences, Oslo and Akershus University College, PO box 4 St. Olavs plass, Oslo, 0130 Norway; 2 Institute of Applied Health Research, School of Health and Life Sciences, Glasgow Caledonian University, Glasgow, UK

**Keywords:** Falls prevention, Home help services, Elderly, Quality of life, Older adults, Exercise, Balance, Preventative care

## Abstract

**Background:**

Falls and fall-related injuries in older adults are associated with great burdens, both for the individuals, the health care system and the society. Previous research has shown evidence for the efficiency of exercise as falls prevention. An understudied group are older adults receiving home help services, and the effect of a falls prevention programme on health-related quality of life is unclear. The primary aim of this randomised controlled trial is to examine the effect of a falls prevention programme on quality of life, physical function and falls efficacy in older adults receiving home help services. A secondary aim is to explore the mediating factors between falls prevention and health-related quality of life.

**Methods:**

The study is a single-blinded randomised controlled trial. Participants are older adults, aged 67 or older, receiving home help services, who are able to walk with or without walking aids, who have experienced at least one fall during the last 12 months and who have a Mini Mental State Examination of 23 or above. The intervention group receives a programme, based on the Otago Exercise Programme, lasting 12 weeks including home visits and motivational telephone calls. The control group receives usual care. The primary outcome is health-related quality of life (SF-36). Secondary outcomes are leg strength, balance, walking speed, walking habits, activities of daily living, nutritional status and falls efficacy. All measurements are performed at baseline, following intervention at 3 months and at 6 months’ follow-up. Sample size, based on the primary outcome, is set to 150 participants randomised into the two arms, including an estimated 15–20% drop out. Participants are recruited from six municipalities in Norway.

**Discussion:**

This trial will generate new knowledge on the effects of an exercise falls prevention programme among older fallers receiving home help services. This knowledge will be useful for clinicians, for health managers in the primary health care service and for policy makers.

**Trial registration:**

ClinicalTrials.gov. NCT02374307. First registration, 16/02/2015.

## Background

### Older adults and health-related quality of life

Health-related quality of life (HRQOL) is of great interest, both with respect to individuals themselves as well as a primary concern of public health administrations and professionals. The remarkable increase in life expectancy in the twentieth century implies a need to focus on factors capable of promoting a high level of HRQOL into old age. In fact, older adults seem to prefer a high HRQOL more than longevity, and researchers have concluded that the key challenge is to preserve a high level of HRQOL rather than increase length of life [1, 2]. HRQOL is a subjective, multidimensional concept shaped by, but not entirely dependent upon, the effects of disease and treatment [3]. The WHO Quality of life (QOL) group defines QOL as “individuals` perception of their position in life in the context of the culture and value systems in which they live, and in relation to their goals, expectations, standards and concerns” [4]. Public health policies in many European countries are therefore primarily concerned with keeping older people living independently in the community with a good quality of life [5–7]. The raise in number of older adults implies more people with chronic diseases and a greater challenge for the health care system in finding effective and feasible interventions to reach this goal [5–8].

Aiming to enable older people to live at home as long as possible, the municipalities in Norway are responsible for providing services in the form of home help for older people [9]. Home help includes services that assist instrumental activities of daily living (iADL), such as vacuum cleaning, and personal activities of daily living (pADL), such as getting dressed, safety alarm services to provide assistance if they fall, and social support. The most important predictor of home care use seems to be dependency in IADL and ADL and cognitive impairment [10]. Home help receivers constitute a transitional group between independent community living older people, and people living in residential care facilities/nursing homes [11]. The combination of the increase of older people and the use of the so-called LEON (“lowest most efficient level of care”) principle [6], makes this a steadily growing group in society, and can be seen as an especially vulnerable group among the older population. Moreover, as economic resources are scarce, there seems to be more focus on post-acute care instead of health promotion and prevention to maintain older adults at home [12]. To date there is no evidence-based practice standard for falls-prevention in Norwegian home care services.

### Older adults and falls

Falls and fall-related injuries are common in older adults and are associated with substantial economic costs that are borne by individuals, the community, and the medical system as a whole [13, 14]. Up to 40% of all nursing home admissions have been found to relate to falls and instability [15]. Important risk factors for falling in the group of older adults are impaired balance and gait, polypharmacy and a history of falls [16]. Poor nutritional status has also been associated with an increased risk of falling, [17] and malnutrition or being in risk of malnutrition is prevalent in half of the older adults receiving home care services [18]. Common consequences of a fall are fear of falling, activity restrictions, loss of mobility and loss of independence [19]. Falling**,** or being at risk of falling also has a negative influence on QOL [20]. Hence, it can be argued that HRQOL is an important outcome in the assessment of falls-prevention programmes [21].

After several decades of research on interventions to reduce falls and fall risk factors, there is now strong evidence for the effectiveness and cost-efficiency of exercise in reducing the number of falls [14, 22–25]. An important, but yet understudied group when it comes to the effect of falls prevention programmes are older adults receiving home help services, and especially those who recently have experienced a fall [11]. Previous research has shown that falls and fear of falling are common in this population and are correlated with the amount of home care needed [11, 26]. Vikman et al. [11] concluded that future studies should have a focus on the effects of falls prevention programmes in the group of those receiving home help services. Recently it has been shown that home help receivers fell more frequently than the independent home-dwelling older population [27]. Low functional level and high home care recipient health problems were independently associated with risk of falling [27]. Fear of falling is also reported more frequently in the group of older adults receiving home help services compared to those who do not receive home care [28]. This suggests that the higher level of fear of falling could be due to a higher level of frailty in this group. Finally, it has been shown that elderly home help receivers in Sweden have a lower QOL compared to those without help and that QOL was negatively correlated with the amount of help needed [29].

### Interventions to improve quality of life

Although exercise-based falls prevention programmes have shown a clear effect on falls incidence and fall risk factors in the general older population, the evidence is still inconsistent about the effects on HRQOL and in particular related to the population of home help receivers [11, 21]. A systematic review by Vaapio et al. [21] considered the specific effect of falls prevention programmes on QOL. The review looked at 12 RCTs including older adults, but none of the studies were aimed at home help receivers. Six of these studies showed a positive effect on QOL. The interventions in these studies ranged from exercise (two studies), information based (one study), to comprehensive geriatric assessment (one study). The review concluded that there is a lack of evidence about the potential benefits of falls prevention programmes on QOL in older people and that more research is needed.

To the authors’ knowledge, only two RCTs have been examining exercise interventions aimed specifically at the population of older home help receivers [30, 31]. The first study tested a home-exercise programme and found positive results on maximum walking speed, but unfortunately the assessors were not blinded to the intervention [31]. The other study explored the effects and costs of a multifactorial, interdisciplinary team approach to falls prevention in 109 older home help receivers with a risk for falls [30]. Exercise was part of the programme, but the amount and mode of exercise varied according to individual needs. At 6 months, no difference in the mean number of falls between groups were found. Subgroup analyses showed that the intervention effectively reduced falls in men (75–84 years old) with a fear of falling or negative fall history, but it is unclear whether the study had sufficiently power for subgroup analyses [30]. Nevertheless, the secondary outcome of QOL significantly improved in the intervention group.

The effect of exercise interventions on HRQOL in the general older adult population have had mixed results, reporting both statistically significant positive effects as well as no significant changes [32–35]. A meta-analysis found no difference between aerobic and strength training, suggesting that the different exercise modes yielded the same effect on self-reported physical function domains of HRQOL [34]. Acree et al. [3] concluded that healthy older adults who regularly participated in physical activity of at least moderate intensity for more than 1 h per week had higher HRQOL measures in both physical and mental domains than those who were less physically active. Although many intervention trials have found a positive association between exercise and HRQOL, the available data from other intervention trials conducted among older adults is inconsistent. Additionally, information of the most effective mode of exercise that may influence HRQOL is lacking [32–35]. Self-efficacy is a possible psychological mediating factor and physical function is a possible physiological mediating factor. Previous research has shown that self-efficacy beliefs can be related to well-being following exercise interventions [36, 37] and that self-efficacy can explain adherence to exercise programs [38–41]. A central concept of the self-efficacy theory is so-called performance accomplishment, i.e. mastery experiences related to certain activities [42], and this points towards testing the mediating effect also of physical function.

The primary aim of this study is to explore the effects of a falls prevention programme, lasting 12 weeks, on HRQOL in older adults receiving home help services. Effects on the secondary outcomes, physical function and falls efficacy, will also be explored. A secondary aim of this study is to explore the mediating factors between falls prevention and HRQOL.

## Methods

### Study design

The study is a single-blinded, pragmatic RCT comparing one intervention group with a control group. The intervention group will receive an adapted version of the Otago Exercise Programme (OEP) over 12 weeks, while the control group receives usual care. Measurements are performed at baseline, at 3 months and at 6 months. The intervention and assessments will be conducted in the participants’ homes. Assessors will be blinded to group participation.

### Study setting and recruitment

**Fig. 1:**
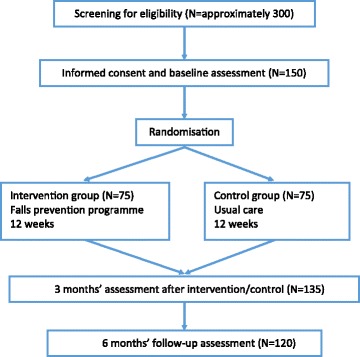
Planned flow of participants in the study

Six municipalities in the Oslo region have agreed to take part in the research project. Participants are recruited through consultants in the municipalities coordinating and providing home help services. The researcher visits the municipalities on a regular basis to conduct the recruitment. Additionally, health workers in the municipalities are informed about the criteria to participate and will alert about eligible participants. Eligible participants will be contacted by the researcher by telephone and asked to consent to being sent information about the study. After a week, they will be contacted again to see if they consent orally to participate. Before baseline testing, the participants must provide a written informed consent. Figure 1 presents the planned flow of participants in the study.

### Inclusion and exclusion criteria

Inclusion criteria are: Individuals who 1) are 67 or older, 2) receive home help services 3) have experienced at least one fall during the last 12 months, 4) are able to walk with or without a walking aid and 5) understand Norwegian. Exclusion criteria are: 1) medical contraindications to exercise, 2) life expectancy below 1 year, 3) a score below 23 on the Mini Mental State Examination (MMSE) and 4) currently participating in other falls prevention programmes or trials.

### Randomisation

The participants are randomly assigned at a 1:1 ratio to the intervention group and the control group. A computer-generated, permuted block randomisation scheme is used to allocate the participants. Following randomisation, the participants receive information by telephone on which group they are allocated to. See flow chart in Fig. 1.

In order to optimize the rigor of the RCT and to minimize bias, a number of methodological factors have been incorporated into the design of the study. The study participants will be randomly allocated to the groups via concealed allocation, as inadequately concealed allocation has been associated with bias in RCTs [43]. Due to the nature of the intervention, it is not possible to blind the participants or the treating therapists to the allocated groups. However, all assessors are blinded to the allocated groups. In further attempts to reduce bias, data will be analysed on an intention-to-treat basis. This preserves the randomisation process and imitate the real-life situation where the possibility exists that not all participants receive the prescribed treatment.

### Study intervention

The intervention performed is based on the OEP, including home visits and motivational phone calls [44]. Balance exercises comprise tasks in standing, walking backwards, stair-walking and rising from a chair. Strengthening exercises uses ankle weight cuffs to strengthen hip extension and abduction, knee flexion and extension and ankle plantar and dorsiflexion. The programme also includes warm-up exercises as movement of neck and shoulders. The OEP has been described in more detail previously [44]. This programme has been shown to be effective in reducing number of falls and number of injuries resulting from falls in addition to improving strength and balance, and maintaining falls efficacy in home-dwelling older adults [45].

In previous studies the OEP has been performed over a period of 1 year [45]. A meta-analysis by Sherrington et al. [46], looking at the effect of falls prevention programmes, recommend a dose of at least 3 hours of exercise weekly for 6 months. This weekly dose is attempted, but the duration of 3 months is shorter than in previous studies. Nevertheless, as in the original OEP, the same number of home visits and telephone calls will be made, and the participants will be encouraged to do a sufficient amount of exercise between home visits. The rational for the change in duration and frequency is both theoretical and pragmatic. Participants included for this study are frail older adults who have a fall history and who receive home help services. Previous research has shown that home help receivers fall more frequently and have a higher level of fear of falling [27, 47]. Poor health, fear of falling, depression and lack of strength are barriers for older adults in order to adhere to exercise programmes [48]. The participants in this study are thus likely to have a lower level of observance compared to more independent elderly and a duration of 1 year might be too long. Additionally, previous research has shown that falls prevention programmes which were considered too demanding by the participants even had a negative impact on QOL [49]. On the other hand, only receiving a few visits might not provide sufficient support which in turn could limit adherence. The pragmatic rationale relates to the organizational structure of physiotherapy services in the primary health care. For this group of older adults an intervention of 3 months is within the time frame of what the physiotherapists normally can provide. Finally, previous research has shown that also falls prevention programmes with a shorter duration than 6 months have had a positive effect on QOL [21].

The physiotherapists visit the participants at home five times during the intervention (week 1, 2, 4, 8 and 10) for instruction and for guiding the appropriate level and progression of each exercise. This includes one additional visit compared to the first 12 weeks of the original OEP intervention [44]. Each visit will take about 1 hour. The first visit may take longer is initial information is given and a relationship is established. At this visit advice related to safety when performing exercises is provided to the participants, both orally and written. In between supervised sessions, participants will be encouraged to continue exercising on their own three times weekly for 30 min. Equipment for exercising (ankle cuff weights of 1, 1,5, 2 and 2,5 kg) is provided for each participant. The weeks between home visits, the physiotherapists call the participants to motivate them to continue exercising and to answer possible questions. As a part of the programme, the participants are also encouraged to perform at least two or more weekly walks of ≥30 min. The participants are provided with a written exercise booklet including illustrations. Following the intervention period, the participants to keep the exercise equipment and booklets, in order to continue exercising.

The participants in the control group will receive usual care from the primary health care service. Following reassessment, the participants will have opportunity to participate in other falls prevention programmes, for example, already existing balance exercise group classes.

### Education of intervention deliverers

Workshops and meetings will be held to inform the physiotherapists participating in the project. Before starting recruitment, one full day workshop on falls prevention and OEP is held for all therapists. Following start-up of recruitment and until the end of the project one workshop will be held approximately every forth months. These last half a day and include one lecture on a topic concerning older people and time for discussion on the development of the project. Additional to the workshops, the researcher will have monthly meetings with the physiotherapists in the different municipalities. In order to make sure that the intervention is performed as intended, a fidelity checklist based on the OEP-manual has been developed. The physiotherapists use the checklist when conducting the home visits and phone calls.

### Outcome measures

Following recruitment participants are assessed before they are randomised. Assessors are blinded to the participants´ group assignment. The time window between baseline assessment and start of intervention is aimed to be within 2 weeks, and the same time window for assessments due at three and 6 months. Measurements and their order are selected to avoid physical and mental fatigue of the participants. Outcome measures that are employed have established reliability and validity, as recommended by the CONSORT group [50]. In addition to improving measurement quality and outcomes, it enables direct comparisons with other studies that investigate HRQOL and can possibly contribute to meta-analyses.

At baseline the Mini Mental Statement Examination (MMSE), a measurement of “Global cognitive function”, is performed and is used as exclusion criteria. The maximum score is 30. A score below 23 indicates cognitive impairment and these participants are excluded [51]. Sociodemographic characteristics, like age and education, are also assessed at baseline. Primary and secondary outcome measures will be performed at baseline, at 3 months and at 6 months’ follow-up.

### Primary outcome variable

*HRQOL* is the primary outcome measured by the Short Form 36 Health Survey (SF-36) [52]. This is a generic and validated questionnaire which, translated into Norwegian, is conducted as an interview [53]. The 36 items in SF-36 are grouped into eight health status scales: physical functioning, role limitations due to physical problems and due to emotional problems, bodily pain, general health perception, vitality, social functioning and mental health [52].

### Secondary outcome variables

In addition to the SF36, the EQ-5D (1990 EuroQOL EQ-5D) is reported. The EQ-5D is a generic and validated questionnaire [54–57]. It describes five dimensions of HRQOL (mobility, self-care, usual activities, pain/discomfort, anxiety/depression), each of which can take one of five responses at five levels of severity (no problems/slight problems/moderate problems/severe problems/severe problems/extreme problems).

*Physical function* includes measures of balance, gait speed, muscle strength as well as activities of daily living. The Bergs Balance Scale is a 14-item scale, which is applied to assess static and dynamic balance in older adults [58]. Gait speed is assessed by measuring usual walking speed over four meters [59] and muscle strength is measured by the 30 s sit to stand test [60]. Instrumental ADL is recorded using the Norwegian Version of the Lawton IADL scale, which is a valid and reliable measure of a person’s self-reported ability to perform complex activities of daily living [61].

*Physical activity* is measured using the “Walking habits questionnaire”, a valid questionnaire for walking habits and physical activity for frailer older people [62]. This questionnaire assesses general behaviour of walking, regarding how often and for how long. The following questions are asked: “Do you take a daily walk?” (yes/no) or “If you do not take a daily walk how many times per week do you take a walk?” (never/almost never/1–2 days/3–4 days/ almost daily) and “How long does you walk generally last? (0–15 min/15–30 min/30–60 min/ 1 h–2 h/>2 h)”. Walking time in minutes per week is calculated by taking the lowest level of days multiplied by lowest level of minutes for each response alternative [62].

*Nutritional status* is measured using the Mini Nutritional Assessment (MNA- elderly, Société des Produits Nestlé, S.A., Vevey, Switzerland) form. The first screening part of six questions is used which includes measurement of weight and height for calculation of BMI [63–65].

*Falls efficacy* is assessed using the Falls Efficacy Scale International (FES-I). This scale has shown good reliability and validity assessing concerns about falling in older adults, and is recommended for clinical trials and practise [66, 67]. It is a self-reported questionnaire, containing 16 items on different activities of daily living. Level of concern is measured on a four-point scale ranging from 1, which is not at all concerned, to 4 which is very concerned [68].

Adherence to the programme is documented through an activity diary completed by the participants and a form checked by the physiotherapists during home visits and calls. Additionally, the participants have a falls calendar where they report adverse events. Adverse events are registered in the following four categories: falls, cardiovascular events, musculoskeletal injuries and health care utilization and will be documented as “due to the intervention” or “not due to the intervention” [69].

### Sample size estimation

The sample size is estimated from the primary outcome, HRQOL (SF-36). A treatment difference of 10 points between the two groups in one of the domains in SF-36 is regarded to be of statistical and clinical significance. The associated standard deviation is assumed to be around 20 points. This implies a moderate effect size [70], which can be expected as previous OEP studies have shown substantial effects on physical outcomes [45]. Moreover, a similar Norwegian study, which included older adults performing exercise following discharge from hospital, estimated the required sample size identically [71]. Given a power of 80% and level α = 0.05, we aim at including 150 participants, allowing for a 15–20% dropout, to detect a difference of 10 points between groups (see Fig. 1).

### Statistical procedures

Statistical analysis is performed using SPSS or a similar statistical package. Descriptive data are reported for variables of interest. The data will be analysed following the intention to treat principle [72]. Prospective differences in primary and secondary outcomes and baseline characteristics between the intervention group and the control group will be assessed by t-tests for continuous and normal distributed variables and with non-parametric tests for categorical variables. Multiple linear regression modelling are used to control for confounding of between-group differences [73]. Hypotheses about mediating factors are tested through correlations, multiple regressions and bootstrapping methods exploring the correlations and explained variance of the chosen mediating factors and the changes in QOL [74]. Bootstrapping is a non-parametric method and is considered favourable with dichotomous variables (group 1 and 2) and small samples (*n* < 250) [74].

## Discussion

The main purpose of the study is to evaluate the impact of OEP on HRQOL in older adults receiving home help services. We anticipate that the intervention described in this protocol will have a positive impact on the HRQOL. The tailored intervention will have a potential to promote evidence-based decision-making and empower older people receiving home help services to remain to a greater extent in charge of their own lives. We rely on a systematic approach, which corresponds with the guidance on developing and evaluating RCTs [75]. Only a few studies have included HRQOL when measuring the effect of a falls prevention programme, and most of these studies include it as a secondary outcome [21, 76]. Outcomes examining HRQOL are selected based on literature identifying a standard set of measurements in falls prevention programmes [77, 78]. SF-36 is chosen due to its good validity, reliability and responsiveness when assessing older adults [79]. This outcome is detailed and broad, but it might be putting a burden on the participants due to its length and sensitive questions. Nevertheless, estimating HRQOL is important to determine whether the effect of a falls prevention programme is significant enough to achieve clinical relevant changes and thus to justify the implementation.

Several studies have looked at the effect of exercise on HRQOL, but to the authors’ knowledge, none of them have specifically focused on older adults who receive home help services and who have a fall history. Studying the relationship between exercise and HRQOL is interesting due to the potential influence of exercise on both health and wellness through improvement of HRQOL [3, 80]. However, results from previous clinical exercise trials have reported mixed effects on HRQOL following exercise [32–35, 81]. Although many studies have found a positive association between exercise and HRQOL, available data from other trials is inconsistent and lacks information on the most effective mode of exercise that may influence HRQOL [32, 33, 71, 81]. This study can provide insight into the effect of falls preventative exercise and its applicability to home-dwelling older fallers with dependency of help from the primary health care service.

There are two ways an intervention mechanism can influence HRQOL, it can be a mediator or a moderator [82]. A mediating factor is defined as an intervening causal factor that may provide information concerning why the intervention increases HRQOL. Moderator mechanisms help us to understand for whom an intervention works [38] and can be classified as either characteristic of the person/group i.e. baseline characteristics or characteristics of the exercise protocol [38, 83]. Mediating mechanisms between HRQOL and falls-prevention programs may be both physiological, such as increased balance and strength, and psychological, such as self-efficacy [38]. A recent study provide evidence that fear is related to falls and concluded that falls self-efficacy plays a mediation role on the relationship between fear of falls and falls [84]. They recommend that any falls prevention should consider psychological covariates of falls, especially subjects’ self-efficacy to reduce falls, alongside other risk factors and covariates of falls. More theoretically driven research on these mechanisms behind treatment effects have been recommended [85–87].

It is widely accepted that falls and subsequent injuries are likely to result in a substantial reduction in quality of life for the persons affected as well a substantial economic burden to the healthcare system [88]. This provision of OEP in this setting could potentially be a beneficial and cost-effective intervention for this group of frail older adults, just as it is for community-dwelling older adults. Several studies have performed analysis on cost-effectiveness of exercise programmes which have shown that it can reduce healthcare costs [14, 89]. Due to its large sample size and theoretically based intervention the present study has the potential to generate new knowledge that may improve the design of future activity programmes for older fallers receiving home help services. Since both outcome measures as well as the intervention are carried out in a clinical setting, relevance and application of study findings to clinicians is enhanced. Results from this study will be primarily of interest to, and could be used by, health care managers and clinicians. Particularly, the results will be useful in decision making to set priorities relating to prevention measures in the community, to appropriately allocate resources and to assess costs and benefits of a falls prevention programme. Finally, the results can be useful for policy makers, in order to put preventative healthcare for this group of frail older adults on the agenda.

To conclude, older people receiving home help services represent a growing and diverse group as part of the population of community-dwelling older adults. The appropriate assessment of HRQOL, the mechanisms behind the relationship between fall prevention and HRQOL, the most effective mode of exercise, as well as the clinical relevance of the results, are challenging issues which are important to address.

## Abbreviations

ADL: Activities of Daily Living
BMI: Body Mass Index
EQ-5D: European Quality of Life - 5 Dimensions
HRQOL: Health-related Quality of Life
iADL: Instrumental Activities of Daily Living
LEON: Lowest most efficient level of care
MMSE: Mini Mental State Examination
MNA: Mini Nutritional Assessment
OEP: Otago Exercise Programme
pADL: Personal Activities of Daily Living
QOL: Quality of Life
RCT: Randomised Controlled Trial
SF-36: Short Form 36 Health Survey
WHO: World Health Organization
